# Macrophages and Fibroblasts, Key Players in Cancer Chemoresistance

**DOI:** 10.3389/fcell.2018.00131

**Published:** 2018-10-09

**Authors:** Lucy V. Ireland, Ainhoa Mielgo

**Affiliations:** Department of Molecular and Clinical Cancer Medicine, University of Liverpool, Liverpool, United Kingdom

**Keywords:** macrophages, fibroblasts, tumor stroma, tumor microenvironment, chemoresistance, therapy resistance

## Abstract

Chemotherapy is routinely used in cancer treatment to eliminate primary and metastatic tumor cells. However, tumors often display or develop resistance to chemotherapy. Mechanisms of chemoresistance can be either tumor cell autonomous or mediated by the tumor surrounding non-malignant cells, also known as stromal cells, which include fibroblasts, immune cells, and cells from the vasculature. Therapies targeting cancer cells have shown limited effectiveness in tumors characterized by a rich tumor stroma. Tumor-associated macrophages (TAMs) and cancer-associated fibroblasts (CAFs) are the most abundant non-cancerous cells in the tumor stroma and have emerged as key players in cancer progression, metastasis and resistance to therapies. This review describes the recent advances in our understanding of how CAFs and TAMs confer chemoresistance to tumor cells and discusses the therapeutic opportunities of combining anti-tumor with anti-stromal therapies. The continued elucidation of the mechanisms by which TAMs and CAFs mediate resistance to therapies will allow the development of improved combination treatments for cancer patients.

## Introduction

The treatment of cancer with chemical substances, known as chemotherapy, is routinely used for cancer treatment because as it circulates throughout the body it targets not only the primary tumor site but also tumor cells that have spread to other organs which are usually missed with surgical intervention or radiotherapy treatment (Eguchi et al., 2008).

The birth of chemotherapy came after the first world war, using nitrogen mustard as an anti-cancer agent in non-Hodgkin’s lymphoma (Gilman, 1963). This agent was non-specific and showed limited effectiveness as patients experienced relapse after a few weeks. However, this discovery triggered investigation into the drug’s mechanism of action leading to the development of other alkylating agents (Haddow et al., 1948). Targeted chemotherapy was developed in the late 1980s after the elucidation of some of the signaling pathways aberrantly regulated in tumors. Targeted chemotherapy included pharmacological targeting of the cell cycle regulating proteins, growth factors and angiogenesis mediators (Hanahan and Weinberg, 2000; Chabner and Roberts, 2005).

Since its beginning, chemotherapy has provided a plethora of benefits for many cancer patients (Klastersky and Paesmans, 2001; Benedetti-Panici et al., 2003; Gebski et al., 2007). Chemotherapeutic agents given before surgery as ‘neoadjuvant’ therapy can be used to reduce the tumor mass before surgical resection. This has many benefits as the reduction of the tumor size decreases the level of invasiveness required for resection and often improves the distinction between healthy and neoplastic tissue during resection (Hayes and Schott, 2015). Adjuvant administration of chemotherapy occurs post-surgery with the purpose of minimizing the chance of recurrence. Adjuvant therapy is effective in two ways: firstly, against micro or macro-metastasis which are already seeded but were not detectable at the time of surgery, and secondly, against micro-metastasis created as a by-product of surgery due to tissue regeneration promoting cytokine storms released after invasive surgery (Hayes and Schott, 2015).

Despite the development of targeted agents with improved toxicity profiles, in some cancers chemotherapeutic agents only provide a minimal improvement of overall survival (Burris et al., 1997; Marquette and Nabell, 2012). The reduced effectiveness of chemotherapy in patients is due to tumor resistance mechanisms, which can be either tumor cell autonomous and/or mediated by the tumor surrounding non-malignant cells present in the tumor microenvironment (TME) (Joyce and Pollard, 2009; De Palma and Lewis, 2013; Mielgo and Schmid, 2013; Zheng, 2017). Tumor cell autonomous mechanisms of drug resistance have been extensively reviewed before (Zahreddine and Borden, 2013; Housman et al., 2014; Zheng, 2017) so the focus of this review is on the emerging TME-mediated mechanisms of tumor resistance to chemotherapy with a main focus on chemoresistance mechanisms mediated by tumor-associated macrophages (TAMs) and fibroblasts.

The TME describes the complete tumor milieu including the malignant tumor cells and the surrounding tumor stroma. The tumor stroma consists of non-malignant cells including immune cells (macrophages, neutrophils, and T cells), fibroblasts, cells from the vasculature (pericytes and endothelial cells) and extracellular matrix (ECM) proteins (Hanahan and Weinberg, 2011). Accumulating evidence shows that the tumor stroma develops and interacts with the tumor cells, participating in bi-directional tumor-stroma signaling which supports tumor progression, metastasis and resistance to therapy (Hanahan and Coussens, 2012; Quail and Joyce, 2013). The most abundant non-cancerous cell types present in the tumor stroma are TAMs and cancer-associated fibroblasts (CAFs). This review will discuss the various mechanisms, discovered to date, by which TAMs and CAFs support tumor chemoresistance, the controversies and current gaps in this research field and the potential future perspectives.

## Origin of Macrophages and Fibroblasts

### Macrophages

Tissue resident macrophages are a diverse population of cells which perform tissue-specific functions in tissue homeostasis, repair, immunity and angiogenesis (Davies et al., 2013a). Macrophages can originate from three independent sources. Embryonic macrophage populations have been mapped back to two sources: fetal liver-derived monocytes or precursor cells found in the yolk sac (Yona et al., 2013; Mass et al., 2016). In adult tissue, macrophage populations differentiate from hematopoietic stem cells in the bone marrow (Orkin and Zon, 2008).

Once established in adult tissue, macrophages maintain their population via self-renewal in the steady state but increase their rate of proliferation in response to stimuli such as interleukin 4 (IL-4) and colony stimulating factor 1 (CSF-1) (Jenkins et al., 2011, 2013; Davies et al., 2013a). During inflammation, bone marrow-derived monocytes are recruited into the tissue and mature into macrophage populations which act alongside tissue resident macrophages (Shi and Pamer, 2011). These converted monocytes display cell surface markers associated with resident macrophages increasing their responsiveness to IL-4 and IL-3 (Yona et al., 2013; Dal-Secco et al., 2015).

Bone-marrow derived macrophages (BM-DMs) and tissue resident macrophages appear to intermingle and work together to resolve inflammation and promote tissue repair. However, it is currently undetermined if BM-DMs play the exact same role as tissue resident macrophages (Davies et al., 2013b). Bone marrow transplant studies have shown that BM-DMs and tissue resident macrophages share similar characteristics (van de Laar et al., 2016). These similarities have been further confirmed by transcriptome analysis of lung alveolar resident macrophages which revealed different genes expressed in BM-DMs compared to tissue resident macrophages (Gibbings et al., 2015).

### Fibroblasts

Fibroblasts are of mesenchymal origin and dependent on their tissue of origin have a distinct transcriptional profile (Chang et al., 2002). Fibroblasts have never been identified in embryonic tissue but are hypothesized to arise during the epithelial-to-mesenchymal transition (EMT) of the epiblast during gastrulation with the generation of mesoderm tissue (Kalluri, 2016). Virchow (1858) identified cells in adult tissue that, produced collagen, were resistant to apoptosis, and reverted to quiescence upon the completion of tissue development. These cells were later called fibroblasts (Virchow, 1858). Due to the inability to identify fibroblasts in embryonic tissue it remains unknown whether the majority of activated fibroblasts originate from fibrocytes or mesenchymal stem cells (MSCs) in adult tissue (Kalluri, 2016).

Stellate cells are found in the pancreas, liver, lung, and kidney and although stellate cells are similar to fibroblasts, they display some distinctly different functions such as vitamin A storage as retinol droplets in their cytoplasm which is required for cellular homeostasis (Keane et al., 2005; Liu, 2006; Erkan et al., 2010). Quiescent stellate cells usually constitute <10% of the organ where they reside and are found in perivascular and peri-parenchymal regions (Wake and Sato, 1993; Apte et al., 1998; Bachem et al., 1998). Like fibroblasts, the origin of stellate cells is still debated. Neuroectoderm is suggested as a potential origin of pancreatic stellate cells (PaSCs) and hepatic stellate cells (hStCs) (Friedman, 2000). Lineage tracing studies have shown that hStCs can originate from mesoderm in mice, however, lineage tracing studies are currently lacking for PaSCs (Asahina et al., 2009, 2011).

Activated fibroblasts (also known as myofibroblasts) can originate from several different cell types that include quiescent fibroblasts from normal parenchyma, endothelial cells, MSCs, and stellate cells (LeBleu et al., 2013; Kalluri, 2016). For example, the origin of activated fibroblasts which support ductal outgrowth has been disputed in breast tissue. Ucar et al. (2010), reported that miR-212/132 expression in stromal fibroblasts is required to support ductal outgrowth. However, in another study, targeted deletion of miR-212 and miR-132 in embryonic stem cells did not show an effect in ductal outgrowth, instead, this study claims that Hic1 expression in stromal cells is required for mammary ductal outgrowth (Kayo et al., 2014). These contradictory results suggest that further studies aiming to understand the role of fibroblasts in mammary gland development are required (Ucar et al., 2014). The heterogeneous origins of myofibroblasts may play a role in generating populations with different phenotypes and functions. Recent studies have described heterogeneous populations of activated fibroblasts present in pancreatic and breast tumors (Ohlund et al., 2017; Costa et al., 2018) and understanding the functions of these different fibroblast populations in cancer is currently an intensive field of research.

## Physiological Functions of Macrophages and Fibroblasts

Macrophages represent a heterogeneous population of cells that are highly plastic and adapt to their surroundings to perform a variety of functions in tissue homeostasis, repair, and immunity (Wynn et al., 2013). Macrophages respond to tissue-derived or external stimuli adapting their phenotype and function accordingly (Biswas and Mantovani, 2010). A spectrum of different subsets of macrophages with diverse phenotypes and functions co-exist in tissues and the macrophage subsets at the extremes of this spectrum are known as M1 (or classically activated) and M2 (or alternatively activated) macrophages (Murray and Wynn, 2011; Mills, 2012). Macrophages can be polarized into M1-like or M2-like macrophages and their polarization depends on the stimulating cytokine and the length of exposure (Gordon and Martinez, 2010). However, the nomenclature and understanding of macrophage subtypes and functions is still evolving.

**FIGURE 1:**
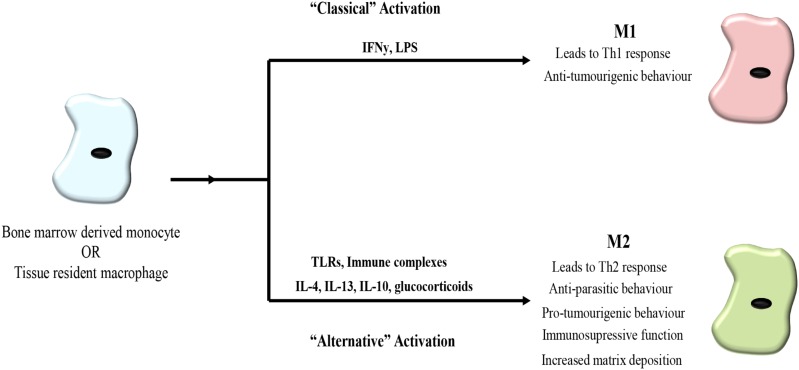
Macrophage polarization. Bone marrow derived monocytes or tissue derived monocytes can be polarized toward either an M1 or M2 phenotype. Classical activation toward M1 polarization occurs in response to interferon gamma (IFNγ) and lipopolysaccharide (LPS) leading to a Th1 response associated with bacteria and viruses as well as possessing anti-tumorigenic properties. Alternative activation toward an M2 phenotype is triggered in response to toll-like receptors (TLRs), immune complexes, IL-4, IL-13, IL-10, and glucocorticoids. M2 macrophages lead to a Th2 response and exhibit anti-parasitic behavior. In cancer, M2-like macrophages promote tumor progression.

M1-like macrophages are generated in response to interferon gamma (INFγ) and lipopolysaccharide (LPS) stimulation, factors produced by infiltrating bacteria and pathogens. M1-like macrophages are pro-inflammatory and secrete factors to promote inflammation, microbicidal activity and immunostimulation, such as cytokines IL-12, IL-6, IL-1β, tumor-necrosis factor alpha (TNFα) as well as reactive oxygen species (ROS) and nitric oxide (NO) (Gordon and Martinez, 2010; Biswas et al., 2013) (**Figure 1**).

In contrast, M2-like macrophages are polarized by IL-4 and IL-13 produced by invading parasites and release anti-inflammatory cytokines IL-10, arginase I and transforming growth factor beta (TGF-β), as well as vascular endothelial growth factor (VEGF), promoting the remodeling of their surrounding tissue. Concurrently, macrophages upregulate expression of scavenging receptors while downregulating receptors and markers associated with antigen presentation (Biswas and Mantovani, 2010; Mantovani and Sica, 2010) (**Figure 1**).

Tissue resident macrophages play a variety of roles in a tissue context-dependent manner. Largely, they participate in functions usually associated with an M2 phenotype including mediating resolution of inflammation, maintaining tissue homeostasis via the removal of debris, supporting angiogenesis and partaking in immune surveillance (Davies et al., 2013a).

Angiogenesis occurs as part of homeostasis throughout life and is tightly regulated by macrophages (Fantin et al., 2010; Outtz et al., 2011). In mouse embryos, microglia (central-nervous system specific macrophages) migrate to the brain and assist in developmental angiogenesis (Arnold and Betsholtz, 2013). In the central nervous system, macrophages promote endothelial tip cell fusion by acting as a chaperone for endothelial cells in vascular development (Fantin et al., 2010). However, it appears that the actions undertaken by macrophages are tissue-dependent as, conversely, macrophages mediate the regression of blood vessels in the developing retina (Lobov et al., 2005; Fantin et al., 2010).

Another homeostatic function of macrophages is the removal of apoptotic and excess cell debris. This function is extremely important in the regulation of hematopoiesis in which macrophages phagocytose excess erythrocytes and neutrophils (Gordy et al., 2011; Klei et al., 2017). When this process was interrupted in mice they suffered severe neutrophilia, splenomegaly, extramedullary hematopoiesis and decreased body weight (Gordy et al., 2011). Macrophages also regulate immune responses through the ingestion of apoptotic cells preventing leakage of cell-death related factors which could promote inflammation (Savill et al., 2002).

In the event of injury or infection, pro-inflammatory macrophages are recruited to the afflicted area and secrete factors including IL-1β, NO, and TNFα as a defense mechanism to kill any invading pathogens (Murray and Wynn, 2011). The release of these factors can also result in secondary damage to host tissue. To limit the impact of this damage, macrophages either undergo apoptosis or reprogram toward an anti-inflammatory M2-like phenotype (Murray and Wynn, 2011). However, when this process goes awry, and macrophages maintain their pro-inflammatory functions, chronic inflammation occurs and becomes the basis of some auto-immune diseases such as Crohn’s disease, rheumatoid arthritis and autoimmune hepatitis (Sindrilaru et al., 2011; Navegantes et al., 2017). Along with mediating the immunity side of wound healing, macrophages alter their secretory phenotype after inflammation subsides, to promote tissue regeneration. To promote the closure of the wound, macrophages attract and activate fibroblasts through the secretion of TGF-β (Khalil et al., 1989; Murray and Wynn, 2011).

**FIGURE 2:**
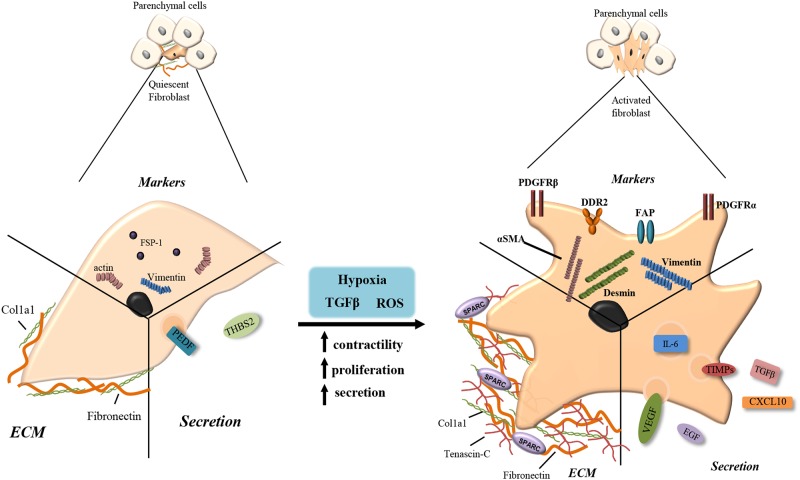
Fibroblast activation. Quiescent fibroblasts produce few extracellular matrix (ECM) components such as fibronectin and collagen type 1 (Col1a1). They express fibroblasts specific protein-1 (FSP-1), actin and vimentin and secrete pigment epithelium-derived factor (PEDF) and thrombospondin-2 (THBS2). When stimulated with transforming growth factor beta (TGF-β), reactive oxygen species (ROS) or hypoxia, quiescent cells become activated increasing their contractility, proliferation and secretion. Activated myofibroblasts produce larger volumes of fibronectin and collagen along with tenascin-c and secreted protein acidic and rich in cysteine (SPARC). Increased secretion includes IL-6, tissue inhibitor of metalloproteinase (TIMPs), TGF-β, vascular endothelial growth factor (VEGF), epidermal growth factor (EGF), and CXCL10. Upregulated receptors/markers include alpha smooth muscle actin (αSMA), platelet derived growth factor receptor alpha/beta (PDGFRα/β), fibroblast activation protein (FAP), discoidin domain-containing receptor 2 (DDR2), desmin, and vimentin.

In healthy tissue, fibroblasts and stellate cells exist in a quiescent state within the ECM making few cell-cell or cell-basement membrane connections. They are usually found as single cells, elongated and spindle-like in morphology situated in the interstitial space between the functional tissues of adult organs (Tarin and Croft, 1969). Quiescent fibroblasts and stellate cells produce very little ECM components such as collagen 1 and fibronectin and secrete a few factors including pigment epithelium-derived factor (PEDF) and thrombospondin-2, although their actual role while quiescent is yet to be fully elucidated (Tarin and Croft, 1969; Pollina et al., 2008). Specific markers for fully quiescent fibroblasts are not yet known, however, fibroblasts specific protein-1 positive (FSP1^+^) cells are often considered as quiescent (Strutz et al., 1995) (**Figure 2**).

The activation of fibroblasts and stellate cells is triggered in response to stress factors produced during tissue stress and damage, including TGF-β and ROS (Kalluri and Zeisberg, 2006). Activated fibroblasts acquire smooth muscle-like properties with increased contractility, motility, proliferation and a stellate morphology, and are known as myofibroblasts (Sappino et al., 1988; Ronnovjessen and Petersen, 1993). Upon activation, stellate cells also acquire a myoblastic phenotype but lose their cytoplasmic retinol lipid droplets (Blaner et al., 2009). Common markers of myofibroblasts include alpha-smooth muscle actin (αSMA), platelet derived growth factor receptor beta (PDGFRβ), PDGFRα, fibroblast activated protein (FAP), vimentin, desmin, Fibronectin Extra-domain A (EDA-FN) and discoidin domain-containing receptor 2 (DDR2) (Ronnovjessen and Petersen, 1993; Sugimoto et al., 2006; Quail and Joyce, 2013; Kalluri, 2016; Jiang et al., 2017) (**Figure 2**).

Myofibroblasts classically function in acute wound healing, becoming ‘reversibly’ activated and depositing the ECM proteins, collagens and fibronectin to close the wound (Dvorak et al., 1986; Darby and Hewitson, 2007). Myofibroblasts also modulate ECM consistency secreting matrix metalloproteases (MMPs) and tissue inhibitor of metalloproteinase (TIMPs) (Tampe and Zeisberg, 2014). Activated myofibroblasts also possess an altered secretory phenotype producing factors such as TGF-β, VEGF, C-X-C motif chemokine ligand 10 (CXCL10), CXCL12, IL-6, and epidermal growth factor (EGF) to promote proliferation and mediate recruitment of other cell types to the damaged tissue (Dvorak et al., 1986) (**Figure 2**).

Chronic activation of fibroblasts and stellate cells occurs in response to prolonged afflictions including toxins or auto-immune disorders. This results in chronic tissue fibrosis with myofibroblasts continuing to aberrantly perform their wound healing functions without resolution. These myofibroblasts become fibrosis-associated fibroblasts (FAFs), are irreversibly activated and exhibit enhanced proliferation and survival (Rock et al., 2011; Zeisberg and Zeisberg, 2013; Kalluri, 2016).

## Tumor-Associated Macrophages (TAMs) and Cancer-Associated Fibroblasts (CAFs)

Macrophages and fibroblasts are the two most abundant non-cancerous cells in tumors. Tumors become infiltrated with BM-DMs that are attracted to the tumor via the secretion of damage associated molecular patterns (DAMPs) and specific macrophage chemoattractants CSF-1 and chemokine C-C motif ligand 2 (CCL2). M1-like macrophages derived from the bone marrow and tissue resident macrophages are recruited to and activated in the tumor site in response to antigen presentation and inflammatory responses (Zhu et al., 2017). Once the tumor is established, tumor cells secrete cytokines IL-4, IL-10, IL-13 and lactic acid, and along with the presence of CD4^+^ Th2 cells, cause the polarization of TAMs toward an M2-like phenotype. The M2 TAMs no longer serve to destroy the tumor but rather support cancer growth, metastasis and resistance to therapies (Gocheva et al., 2010; Qian and Pollard, 2010; Ruffell et al., 2012; Colegio et al., 2014). M2 TAMs support tumor progression by directly stimulating the growth of cancer cells through the production of growth factors, including EGF, TNFα, IL-6 (Grivennikov et al., 2010).

Solid tumors can undergo periods of hypoxia as its growing size limits the disposal of waste products and nutrient delivery becomes limited, triggering the angiogenic switch (Bergers and Benjamin, 2003; Hanahan and Weinberg, 2011). The activation of the angiogenic switch in tumors triggers dysregulated angiogenesis resulting in leaky vasculature with abnormal branching and enlarged diameter (Bergers and Benjamin, 2003). Macrophages are a source of VEGF and are known to support angiogenesis under normal physiological conditions (Fantin et al., 2010; Outtz et al., 2011). However, tumors depleted of myeloid-derived VEGF have a normalized vasculature with increased pericyte coverage and reduced vessel length and this accelerates tumor progression (Stockmann et al., 2008). Conversely, another study showed that hypoxia-related TAMs possess reduced mTOR activation, and that stimulation of mTOR activity in TAMs resulted in normalized vasculature with decreased vessel leakiness, hypoxia and metastasis (Wenes et al., 2016). TAMs are attracted to areas of tumor hypoxia through the release of Semaphorin 3A by cancer cells and TAMs promote angiogenesis via the phosphorylation of VEGF-receptor on endothelial cells (Casazza et al., 2013). CSF-1 stimulation has been shown to upregulate TIE2 expression on macrophages (Forget et al., 2014). Once inside the tumor, TIE2+ macrophages bind to angiopoietin-2 (Ang-2) expressed by endothelial cells and stimulate the growth of blood vessels promoting tumor growth and metastasis (De Palma et al., 2005; Mazzieri et al., 2011).

Metastatic spread of tumor cells to distant organs involves a multi-step process that requires local tissue invasion, intravasation, circulation through the blood stream, extravasation and successful colonization of the distant organ by the cancer cells (Hanahan and Coussens, 2012; Massague and Obenauf, 2016). Macrophages play a role in each of these stages of the metastatic cascade. Macrophages help tumor cell invasion into the basement membrane (Condeelis and Pollard, 2006; Wyckoff et al., 2007). In the PyMT breast cancer mouse model, CSF-1 produced by tumor cells and EGF secreted by TAMs results in the migration of both macrophages and cancer cells along collagen fibers and intravasation into the blood vessels (Goswami et al., 2005; Wyckoff et al., 2007). This phenomenon was also seen in glioblastoma, resulting in enhanced cancer cell invasion (Coniglio et al., 2012). TAMs can also promote tumor cell migration and invasion through the secretion of MMPs, secreted protein acidic and rich in cysteine (SPARC) and cathepsins which degrade and remodel the ECM (Bergers et al., 2000; Gocheva et al., 2006) as well as through the secretion of TGF-β which promotes EMT of tumor cells and increased tumor cell migration (Bonde et al., 2012).

As outlined earlier, fibroblasts are activated in response to tissue damage. After resolution of the insult, fibroblasts will reprogram back to quiescence or undergo apoptosis (Tomasek et al., 2002). However, tumors are referred to as “wounds that do not heal” (Dvorak et al., 1986). Persistent activation signals, in the context of cancer, maintain fibroblasts in a chronically activated state triggering a desmoplastic reaction and generating a dense fibrotic stroma which envelopes the tumor mass. Fibroblast activation signals are tumor-specific and determine the phenotype and function of the resulting myofibroblast. In the TME, a myofibroblast will exert a pro- or anti-tumorigenic response depending upon which chemokines/cytokines it encounters (Tampe and Zeisberg, 2014). TGF-β is a common activating factor released by tumors which increases the expression of PDGF receptors on activated PaSCs (Apte et al., 1999). Sonic hedgehog (Shh) signaling in PDAC tumors has been reported to promote fibroblast activation and fibrosis in the pancreas (Bailey et al., 2008; Yauch et al., 2008). Other common factors involved in CAF activation include fibroblast growth factor (FGF), platelet derived growth factor (PDGF), and monocyte chemotactic protein (MCP1) (Kalluri and Zeisberg, 2006; Marsh et al., 2013).

Concurrent with their activated state, CAFs express an altered secretory phenotype, compared to quiescent fibroblasts, including ECM proteins and ECM modulating factors such as tenascin C, periostin, SPARC and EDA-FN; and tumor promoting factors such as nuclear factor-kB (NF-kB), IL-8, prostaglandin E_2_ (PGE_2_), connective tissue growth factor (CTGF) and CXCL7 (Kalluri, 2003; Hanahan and Coussens, 2012).

Recent advances in the field of CAF research has shown that different subsets of CAF populations with different functions co-exist within tumors (Costea et al., 2013; Brechbuhl et al., 2017; Ohlund et al., 2017; Costa et al., 2018). For example, in PDAC a specific subset of CAFs expressing high levels of αSMA but low levels of IL-6 was found in the fibrotic area juxtaposed to cancer cells and was called the myofibroblast CAF subset (myCAFs) (Ohlund et al., 2017). A different subset of CAFs, expressing low levels of αSMA but high levels of IL-6 was found at the periphery of the tumor and was termed the inflammatory CAF subset (iCAFs) (Ohlund et al., 2017). Ohlund et al. (2017) showed that the proximity of the myofibroblasts to the PDAC tumor cells, and the concentration of tumor-secreted factors alters the phenotype of the CAFs and the proteins they secrete. Another recent study performed with luminal A, human epidermal growth factor receptor 2^+^ (HER2^+^) and triple negative breast cancer (TNBC) patient samples revealed the co-existence of four different CAF subsets in breast tumors (Costa et al., 2018). TNBC samples predominantly had two types of myofibroblast-like CAFs; CAF-S1 and CAF-S4 identified by their high expression of αSMA. However, only CAF-S1 defined as CD29^Med^, FAP^Hi^, FSP1^Low-Hi^, aSMA^Hi^, PDGFRb^Med-Hi^, and CAV1^Low^; showed an immunosuppressive role by attracting T lymphocytes and promoting their survival and differentiation into immunosuppressive T regulatory cells (Costa et al., 2018). Thus, CAFs, like TAMs, are a heterogeneous population of cells and uncovering the different CAF populations and their functions in cancer is currently an important area of research.

Tumor-associated macrophages and CAFs take part in a complex interplay and can regulate each other’s functions. For example, cancer cells and myofibroblasts are known sources of VEGF which promotes the accumulation of immune cells including macrophages at the site of fibrosis (Fukumura et al., 1998). VEGF-dependent recruitment and activation of macrophages promotes tumorigenesis, angiogenesis and invasion in skin cancer (Linde et al., 2012). Reciprocally, in liver metastasis of pancreatic cancer, macrophages recruited to the metastatic liver secrete granulin and activate resident quiescent hStCs which subsequently produce periostin supporting the growth of metastatic cancer cells in the liver (Nielsen et al., 2016).

## Mechanisms of Chemotherapy Resistance Driven by TAMs and CAFs

**FIGURE 3:**
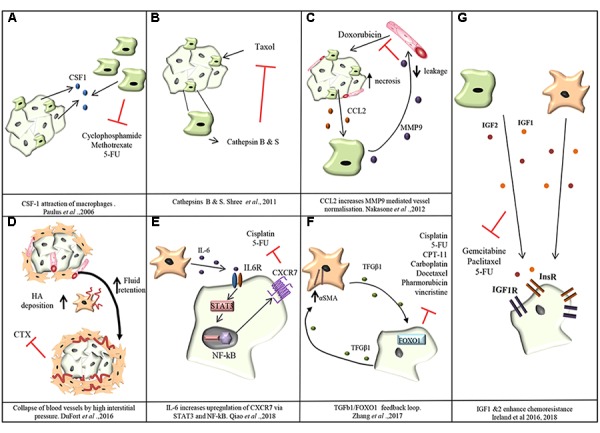
Mechanisms of chemoresistance mediated by TAMs and CAFs. **(A)** Cancer cells attract TAMs via CSF-1. TAMs confer resistance of MCF-7 breast cancer cells toward cyclophosphamide, methotrexate and 5-fluorouracil (5-FU; Paulus et al., 2006). **(B)** Cathepsins B and S secreted by TAMS mediate resistance of breast cancer cells to taxol in MMTV-PyMT mouse model (Shree et al., 2011). **(C)** In the MMTV-PyMT transgenic mouse model, cancer cell necrosis caused by doxorubicin treatment causes cancer cells to release the monocyte chemoattractant CCL2. Recruited TAMs produce MMP-9 which causes leakiness of blood vessels and reduction in doxorubicin delivery (Nakasone et al., 2012). **(D)** In PDAC, CAFs increase deposition of hyaluronan (HA) creating an increase in fluid retention and subsequently interstitial pressure in the tumor rises causing the collapse of blood vessels and limiting the delivery of chemotherapeutic agents (DuFort et al., 2016). **(E)** CAF secreted IL-6 stimulates the upregulation of CXCR7 through STAT3/NF-kB signaling promoting resistance of esophageal squamous cell carcinoma cells against cisplatin and 5-FU (Qiao et al., 2018). **(F)** CAF-derived TGF-β upregulates FOXO1 expression in esophageal squamous cell carcinoma cells triggering reciprocal TGF-β secretion which in turn increases the levels of αSMA expression in CAFs and resistance to cisplatin, taxol, irinotecan (CPT-11), 5-FU, carboplatin, docetaxel, pharmorubicin, and vincristine (Zhang et al., 2017). **(G)** TAM and CAF derived IGF-1 and IGF-2 activate insulin and IGF-1 receptor signaling on tumor cells conferring resistance of pancreatic and breast tumors to gemcitabine and paclitaxel (Ireland et al., 2016, 2018).

Chemotherapy is used as a treatment in many different cancer types and is used either alone or in combination with surgical resection or radiation. Chemotherapy targets tumor cells at both the primary tumor site and the metastatic site. However, a common problem encountered with the treatment of many tumors is an acquired resistance to chemotherapeutic agents. Chemoresistance can be mediated by tumor cell-autonomous mechanisms, including changes in tumor cell epigenetics, drug inactivation, EMT, activation of alternative survival and proliferative pathways, and/or selection of drug-resistant cancer cell clones (Housman et al., 2014). However, many solid tumors such as breast cancer and PDAC have a rich stroma which contains, as described before, a plethora of non-malignant cell types that influence cancer progression and response to therapy in various ways. In fact, these non-malignant stromal cells are not simple bystanders but engage in bi-directional tumor-stroma signaling which can result in impaired therapeutic efficacy. For instance, the attraction of TAMs in a MCF-7 breast cancer xenograft model, via CSF-1 signaling, reduces the efficacy of a combination treatment with cyclophosphamide, methotrexate and 5-fluorouracil (CMF) (Paulus et al., 2006) (**Figure 3A**). The presence of TAMs in the genetic MMTV-PyMT mouse model of breast cancer makes tumors more resistant to paclitaxel therapy (DeNardo et al., 2011). Another study revealed TAM-derived cathepsins B and S as responsible for mediating chemoresistance to taxol in the MMTV-PyMT mouse model (Shree et al., 2011) (**Figure 3B**). In a subcutaneous mouse model of colorectal cancer, IL-6 released by TAMs mediates chemoresistance to 5-FU via activation of the IL-6R/STAT3 signaling axis (Yin et al., 2017).

Tumor-associated macrophages can also regulate the delivery of chemotherapy to tumor cells. In the MMTV-PyMT transgenic breast cancer mouse model, doxorubicin administration causes necrosis of cancer cells with the release of CCL2, a chemokine that attracts monocytes/macrophages. MMP-9 secretion by the recruited myeloid cells was shown to decrease vasculature leakiness and to impair doxorubicin delivery into the tumors (Nakasone et al., 2012). In fact, MMP-9 null mice showed an improved response to Doxorubicin that correlated with increased vascular leakage (Nakasone et al., 2012) (**Figure 3C**). Conversely, in a Lewis lung carcinoma subcutaneous isograft model, myeloid derived VEGF promotes resistance to cyclophosphamide treatment by promoting the formation of abnormal vessels with reduced pericyte coverage, tortuosity, and vessel density (Stockmann et al., 2008).

Cancer-associated fibroblasts also play a role in tumor chemoresistance. In fact, a dense fibrotic stroma correlates with a poor response to neoadjuvant treatment with 5-fluorouracil, epirubicin and cyclophosphamide (FEC) in breast cancer and with gemcitabine in PDAC (Farmer et al., 2009; Olive et al., 2009; Pandol et al., 2009). One way fibrosis promotes chemoresistance in PDAC is through CAF secretion of hyaluronan, generating high interstitial pressure within the tumor, causing the collapse of blood vessels supplying the tumor mass and impairing drug delivery (DuFort et al., 2016) (**Figure 3D**).

In esophageal squamous cell carcinoma, CXCR7 expression is upregulated in tumor cells through STAT3/NF-kB signaling stimulated by CAF-derived IL-6, ultimately promoting resistance against cisplatin and 5-fluorouracil (Qiao et al., 2018) (**Figure 3E**). IL-6 has pleiotropic effects in the TME and also mediates chemoresistance by promoting EMT of cancer cells (Shintani et al., 2016). TGF-β secretion by CAFs was shown to confer resistance of esophageal squamous cell carcinoma against cisplatin, taxol, irinotecan (CPT-11), 5-fluorouracil (5-FU), carboplatin, docetaxel, pharmorubicin, and vincristine (Zhang et al., 2017) (**Figure 3F**).

We recently showed that TAMs and CAFs are the main sources of Insulin-like growth factors 1 and 2 (IGF-1, IGF-2) in pancreatic and breast tumors, and that IGF signaling mediates resistance of murine pancreatic and breast tumors to gemcitabine and paclitaxel (**Figure 3G**) (Ireland et al., 2016, 2018). Importantly, we found that 72% of PDAC patients and 87% of patients with invasive breast cancer have the IGF signaling pathway activated in their tumors, and this correlates with an increased number of TAMs and CAFs (Ireland et al., 2016, 2018). Similarly, IGF1 was also shown to be secreted by TAMs in glioblastoma multiforme and to mediate resistance to a CSF-1R small molecule inhibitor through activation of PI3K signaling (Quail et al., 2016).

## Targeting TAMs and CAFs in Cancer

Currently, approaches are being undertaken to block macrophage recruitment to the tumor site, to repolarize TAMs back into an M1-like anti-tumorigenic phenotype, and to target specific tumorigenic functions of TAMs. Preventing recruitment of macrophages to the tumor site has been achieved through targeting macrophage chemoattractants such as CSF-1 and CCL2 or their corresponding receptors: CSF-1 receptor (CSF-1R) and C-C chemokine receptor type 2 (CCR2). Anti-CSF-1R agents have been shown to be effective against recruitment of M2-like macrophages in breast cancer models, and anti-CSF1R inhibitors used in combination with paclitaxel decreased tumor growth and pulmonary metastasis (DeNardo et al., 2011). CSF-1R and CCR2 antagonists have been reported to prevent infiltration of TAMs into the tumor mass increasing response to gemcitabine treatment in mouse models of PDAC (Mitchem et al., 2013). CCL2 inhibition in combination with docetaxel has shown increased efficacy, compared to docetaxel treatment alone, resulting in decreased tumor growth and metastatic spread in prostate cancer (Loberg et al., 2007). This combination has also shown promise in lung cancer, breast cancer metastasis, and PDAC (Lu and Kang, 2009; Fridlender et al., 2011; Kalbasi et al., 2017). Due to these successes CSF-1, CCL2, and CSF-1R targeting agents are being investigated in clinical trials in combination with chemotherapy in a range of solid tumors (**Table 1**). However, the targeting of chemokines and cytokines has limitations due to their redundant and promiscuous nature. In fact, chemokines and cytokines can often bind to more than one receptor, and at the same time different cytokines/chemokines can bind to the same receptor and activate the same signaling pathway (O’Shea and Murray, 2008; Turner et al., 2014). In addition, to add more complexity, certain cytokine receptors are expressed by several cell types and as a result, inhibiting the cytokine/receptor affects all cell populations expressing the receptor. This is the case with CSF-1R which is not exclusively expressed by M2-like macrophages but is also expressed by M1-like macrophages, neutrophils, myeloid-derived suppressor cells (MDSCs) and dendritic cells (DCs; Cannarile et al., 2017).

**Table 1:** Summary of combination treatments of chemotherapy and stromal targeting agents.

Molecular target	Treatment combination	Cancer type	Clinical trial	Outcome	Reference
CSF1R	Pexidartinib (PLX3397) (αCSF-lR) + eribulin	Metastatic breast cancer	Phase 1/2 NCT01596751	Ongoing	
	Pexidartinib (PLX3397 αCSF-lR) + paclitaxel	Solid tumors	Phase 1 NCT01525602	ORR: 4/23 (17%) CBR: 14/23 (61%)	Rugo et al., 2014
CSF1	MCS110 (αCSFl) + carboplatin plus gemcitabine	Triple negative breast cancer	Phase 2 NCT02435680	Ongoing	
CCL2	CNTO888 (αCCL2) + DOXIL^®^/Caelyx^®^ doxorubicin HC1 liposome injection CNTO888 + gemcitabine CNTO888 + paclitaxel and carboplatin CNTO888 + docetaxel	Solid tumors	Phase 1 NCT01204996	Hematological complications in >93%	
	CNTO888 + docetaxel	Metastatic resistant prostate cancer	Phase 2 NTC00992186	34% maintained stable disease	
CD40	Dacetuzumab + bortezomib	Relapsed or refractory multiple myeloma	Phase 1 NCT00664898	Completed results not posted	
	Dacetuzumab + R-ICE (rituximab, etoposide, carboplatin, ifosfamide)	Diffuse large B cell lymphoma	Phase IIb NCT00529503	Terminated	
Smo	LDE225 (αSmo) + temozolomide	Medulloblastoma	Phase 3 NCT01708174	ORR: 18.8%	Kieran et al., 2013
IGF	BI 836845 + enzalutamide	Castration-resistant Prostatic neoplasms	Phase 1 NCT02204072	Ongoing	
	BI 836845 + everolimus + exemestane	HR^+^/HER2^-^ advanced breast cancer	Phase 1 NCT02123823	Ongoing	
	MEDI-573 + aromatase inhibitor	HER-2 negative metastatic breast cancer	Phase 2 NCT01446159	Ongoing	

Repolarizing macrophages back into an M1-like tumoricidal phenotype appears an attractive approach as the M2 TAMs are already present in the tumor and repolarization could therefore provide an effective strategy to restore the tumoricidal function of macrophages and prevent cancer progression. This has been investigated using an anti-CD40 antibody in combination with gemcitabine in a genetic KPC (Kras ^LSL.G12D/+^; p53^R172H/+^; Pdx^Cretg/+^) PDAC mouse model and in PDAC patients (Beatty et al., 2011). The administration of an agonist CD40 antibody repolarized TAMs back into an M1-like phenotype leading to an increased response to gemcitabine and reduced tumor burden (Beatty et al., 2011). A phase 1 clinical trial has recently been completed for the use of Dacetuzumab (human anti-CD40 mAb) + Bortezomib chemotherapy in patients with relapsed or refractory multiple myeloma, however, results have yet to be published (**Table 1**).

**FIGURE 4:**
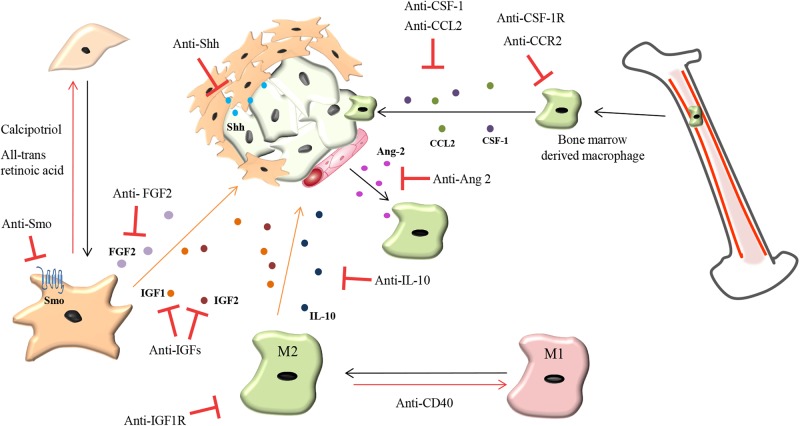
Therapeutic strategies to overcome chemoresistance mediated by TAMs and CAFs. CAFs: Reprogramming activated CAFs back toward a quiescent phenotype by anti-Smoothened (Smo), anti-sonic hedgehog (Shh), all-*trans* retinoic acid and calcipotriol (vitamin D analog) while fibroblast growth factor 2 (FGF2) targeting agents prevents resistance of tumor cells to anti-estrogens in breast cancer. TAMs: Repolarizing M2 macrophages back to an M1-like phenotype can be mediated by a CD40 agonist. Prevention of macrophage recruitment to tumor sites is currently being achieved by targeting the colony-stimulating factor 1 (CSF-1) and C-C motif chemokine 2 (CCL2) signaling axis. Anti-angiopoietin-2 (Ang-2) antibodies prevent TAM interaction with blood vessels. IL-10 produced by TAMs promotes chemoresistance which can be abrogated by treatment with anti-IL-10 antibodies. TAMs and CAFs secrete insulin-like growth factor 1 and 2 (IGF1 and IGF2) which makes pancreatic and breast tumors chemoresistant and more metastatic. Treatment of tumors with anti-IGF blocking antibodies increases the response of pancreatic and breast tumors to chemotherapy and decreases tumor growth and metastasis.

Since TAMs can act as a double edge sword in cancer, with M1-like TAMs exerting anti-tumorigenic functions and M2-like TAMs exerting pro-tumorigenic functions, targeting TAMs pro-tumorigenic functions seems a more promising approach compared to ablation therapies targeting all TAMs. As previously mentioned, TAMs are known to facilitate the intravasation of tumor cells and promote angiogenesis (Wyckoff et al., 2007). Therefore, targeting TAMs role in pathological angiogenesis is an attractive therapeutic opportunity. In MMTV-PyMT mammary carcinomas and RIP1-Tag2 pancreatic insulinomas, an Ang-2 neutralizing antibody administration did not reduce the recruitment of Tie-2^+^ TAMs but instead, prevented their binding to Ang-2 on activated endothelial cells subsequently decreasing angiogenesis and tumor progression (Mazzieri et al., 2011). CSF-1R inhibition increased the efficacy of anti-VEGFR-2 anti-angiogenic therapy in a mouse model of Lewis lung carcinoma (Priceman et al., 2010). M2 TAMs produce IL-10 at the tumor site leading to resistance of breast cancer to paclitaxel treatment (Yang et al., 2015) and this resistance can be abrogated with the administration of an IL-10 neutralizing antibody (Yang et al., 2015) (**Figure 4**).

It is currently unclear whether CAFs play a supportive or restrictive role in tumor progression. Based on the correlation between a large desmoplastic reaction and poor patient outcome it was hypothesized that ablation of the myofibroblasts would improve therapy response and decrease tumor growth. Shh is overexpressed by neoplastic PDAC cells (Thayer et al., 2003), stimulating Gli activity in surrounding fibroblasts and triggering their activation (Tian et al., 2009). Therefore, Shh became a target to inhibit fibroblast activation and Shh inhibition initially showed promising results in a pre-clinical PDAC mouse models. Shh inhibition reduced fibrosis and increased tumor vascularization, improving the delivery of gemcitabine to PDAC tumors (Olive et al., 2009). However, a clinical trial of Saridegib, a Shh inhibitor, with gemcitabine, in metastatic PDAC patients, failed at phase II as patients had reduced survival (Madden, 2012). Further investigation into fibroblast function in PDAC in longer-term experiments with mouse PDAC models showed that fibroblast ablation using smoothened inhibitor or genetic depletion of Shh or αSMA^+^ myofibroblasts, in fact showed that the stroma restrained tumor growth and metastasis (Oezdemir et al., 2014; Rhim et al., 2014). These conflicting results, combined with the emerging evidence that different CAF populations co-exist in tumors, suggest that different CAF populations may have different and possibly opposing effects in cancer progression (Ohlund et al., 2017; Costa et al., 2018). Despite these results, a phase 3 trial in medulloblastoma, using an oral sonidegib (smoothened inhibitor) in combination with temozolomide showed promising results with an objective response rate of 18.8% (**Table 1**).

One approach which warrants further investigation is the reprogramming of the activated CAFs back into their quiescent state. This approach has seen some success in PDAC mouse models using Calcipotriol (vitamin D analog) which reverts myofibroblasts to quiescence, reducing the desmoplastic reaction which in turn improves gemcitabine delivery (Sherman et al., 2014). In 3D models and genetic mouse models of PDAC the use of all-*trans* retinoic acid to restore the quiescence of stellate cells increased vascularity, resulting in increased response to gemcitabine and reduced tumor growth (Carapuca et al., 2016). In estrogen receptor positive breast cancer, CAF-derived FGF-2 promotes resistance to anti-estrogens which is abrogated with administration of an FGF-2 neutralizing antibody (Shee et al., 2018) (**Figure 4**).

As previously mentioned TAMs and CAFs act as stromal sources of IGF 1 and 2 in PDAC, and invasive breast cancer (Ireland et al., 2016, 2018) and this makes tumors resistant to chemotherapy and more metastatic. Blockade of IGF1 receptor signaling in PDAC, using IGF-1R inhibitors, has failed in the clinic (Guha, 2013; King et al., 2014; Gradishar et al., 2016) but appears to be more effective in certain tumor types such as glioblastoma (Quail et al., 2016). In PDAC and invasive breast cancer mouse models, we have shown that both Insulin and IGF1 receptors are activated, and the use of IGF1/IGF2 ligand blocking antibodies, which inhibit IGF-1 and IGF-2 signaling through both IGF-1 and insulin receptors, increases response to chemotherapy and reduces tumor growth and metastasis (Ireland et al., 2016, 2018). These studies suggest that inhibition of signaling through both Insulin and IGF1 receptors by blocking IGF 1 and 2 ligands may be more effective compared to IGF1R inhibitors in certain cancer types which have both receptors activated, such as pancreatic and breast cancer (**Figure 4**). IGF1/IGF2 blocking antibodies are currently being tested in phase I and II clinical trials in patients with castration resistant prostate cancer and metastatic breast cancer patients in combination with chemotherapy (**Table 1**).

## Future Perspectives

Macrophages and fibroblasts are key regulators of tissue homeostasis, repair, angiogenesis and immunity. In tumors, cancer cells, macrophages and fibroblasts co-exist, co-evolve and continuously interact with each other. Tumor cells “hijack” macrophages and fibroblasts to support their own growth and expansion. Specifically, tumors exploit the natural plasticity of macrophages polarizing them into M2-like pro-tumorigenic TAMs that, support tumor growth in numerous ways, as described in this review. The same phenomenon is observed with respect to fibroblast function. Under normal physiological conditions fibroblasts facilitate wound repair by promoting cell growth, migration and ECM deposition. Tumor cells stimulate fibroblast activation and, reciprocally, activated fibroblasts support cancer cell survival, proliferation and resistance to therapies. However, recent findings have shown that different CAF populations with different and possibly even opposite functions co-exist in tumors.

Ablation therapies that eliminate macrophage recruitment to the tumor site have shown some promising results (DeNardo et al., 2011; Mitchem et al., 2013). However, this approach has some limitations, including the lack of specificity for different macrophage subsets and the redundancy of macrophage chemo-attractants. Inhibition of CAFs activation in PDAC patients actually resulted in enhanced tumor progression (Madden, 2012) and CAF ablation therapies in mouse tumor models resulted in increased tumor growth and metastasis (Oezdemir et al., 2014; Rhim et al., 2014). These findings suggest that further investigation into the role of different CAF subtypes is required to design therapies that specifically target defined CAF subtypes and/or functions that support cancer progression. Therapies that specifically target the pro-tumorigenic functions of TAMs and CAFs could lead to a more specific and effective anti-tumor response. To develop specific anti-stroma therapies that only target the pro-tumorigenic functions of TAMs and CAFs, while sparing their anti-tumorigenic functions, we first need to gain a better understanding of the complex composition and function of the tumor stroma.

While TAMs and CAFs are the most abundant stromal cell types in tumors, and as described in this review affect resistance to chemotherapy using a plethora of mechanisms, other stromal/immune cells present in the TME, including MDSCs, DCs, and T cells can also affect the response of tumors to therapies (for reviews/articles on this topic see Castells et al., 2012; Palucka et al., 2013; Son et al., 2017; Weber et al., 2018).

While some key stroma-derived signaling molecules have already been identified, the complex tumor-stroma interactions and the dynamic evolution of these interactions during tumor progression and in response to treatment need to be fully elucidated in order to develop effective anti-cancer therapies with a durable effect.

## Author Contributions

LI and AM co-wrote this review.

## Conflict of Interest Statement

The authors declare that the research was conducted in the absence of any commercial or financial relationships that could be construed as a potential conflict of interest.
